# Associative Bacteria and Arbuscular Mycorrhizal Fungus Increase Drought Tolerance in Maize (*Zea mays* L.) through Morphoanatomical, Physiological, and Biochemical Changes

**DOI:** 10.3390/plants13121667

**Published:** 2024-06-16

**Authors:** Angélica Nunes Tiepo, Mateus Henrique Fávaro, Talita Silveira Amador, Leonardo Fernandes Tavares, Mariana Fernandes Hertel, Anderson Kikuchi Calzavara, André Luiz Martinez de Oliveira, Halley Caixeta Oliveira, Jaqueline Dias-Pereira, Hugo Humberto de Araújo, Edmilson Bianchini, José Antonio Pimenta, Renata Stolf-Moreira

**Affiliations:** 1 Department of Animal and Plant Biology, UEL—State University of Londrina, Londrina 86057-970, PR, Brazil; angelicantiepo@gmail.com (A.N.T.); mateus-favaro@hotmail.com (M.H.F.); talitamador@hotmail.com (T.S.A.); leonardo.fernandes@uel.br (L.F.T.); mariana.f.h@hotmail.com (M.F.H.); halley@uel.br (H.C.O.); bianchini@uel.br (E.B.); pimenta@uel.br (J.A.P.); 2 Department of Biochemistry and Biotechnology, UEL—State University of Londrina, Londrina 86057-970, PR, Brazil; almoliva@uel.br; 3 Institute of Biological and Health Sciences, UFV—Federal University of Viçosa, Rio Paranaíba 36570-900, MG, Brazil; jaqueline.dias@ufv.br; 4 Department of Plant Biology, UFV—Federal University of Viçosa, Rio Paranaíba 36570-900, MG, Brazil; hugo.humberto@ufv.br

**Keywords:** plant–microorganism interaction, water deficit, anatomical parameters, chlorophyll fluorescence, maize crop

## Abstract

Water deficiency has been recognized as a major abiotic stress that causes losses in maize crops around the world. The maize crop is very important due to the range of products that are derived from this plant. A potential way to reduce the damages caused by water deficiency in maize crops is through the association with plant growth-promoting bacteria (PGPB) and arbuscular mycorrhizal fungi (AMF). To define the mechanisms developed by associative PGPB and AMF in maize that are involved in protection against moderate drought (MD), this study evaluated the biometrical, anatomical, biochemical, and physiological parameters of maize grown under MD and inoculated with different PGPB (*Azospirillum brasilense* strain Ab-V5 and *Bacillus* sp. strain ZK) and with AMF. The relative water content did not change in the treatments. The association with ZK increased the shoot:total ratio, total dry weight, maximum quantum yield of photosystem II, vascular cylinder thickness, and vascular cylinder area. The Ab-V5 inoculation led to an increment in root dry weight, the area of metaxylem vessel elements, and nitrate reductase activity. The AMF association did not lead to changes in the measured parameters. The results indicate that the association with PGPB is a relevant alternative to contribute to reducing losses in maize crops under drought. However, AMF is not indicated for this crop under drought.

## 1. Introduction

Water deficiency is a limiting environmental factor for plant growth since changes in physiology, nutrient uptake, and metabolism induced by drought can interfere with the allocation of plant biomass [1]. Thus, water deficiency has been recognized as the major abiotic stress that causes substantial crop losses around the planet [2,3].

Maize is one of the most important crops, as verified by the range of products that are derived from this plant. In 2017, approximately one billion tons of maize were produced globally [4]. However, drought negatively affects the growth and production of this crop [5].

Drought events have increased in recent decades in association with climate changes caused by global warming [6]. In 2012, in the USA, the drought was the worst in 60 years, causing the lowest maize production since 1995 [7]. Moreover, production is associated with the availability of water, especially in the critical period of crop growth, from the pre-flowering to the beginning of the grain filling [8]. These facts highlight the need to develop technologies that reduce crop losses in drought situations.

Therefore, the association with plant growth-promoting bacteria (PGPB) and arbuscular mycorrhizal fungus (AMF) has been applied as a way to improve the tolerance of plants against abiotic stress, like drought [9,10].

The PGPB is characterized by the ability to colonize the plant’s root, where they provide essential nutrients or facilitate their access to plants [1,11]. They still can modulate plant hormone levels through the production of auxins, cytokinins, gibberellins, and abscisic acid and may also modulate the response to ethylene [12,13]. This leads to changes in the development of the root system, which can provide an increase in the absorption of water and essential nutrients [14]. Besides that, the PGPB association can influence enzyme activity, like nitrate reductase (NR), and the reduction in oxidative damage, by increasing the elimination of oxygen reactive species (ROS), both leading to an enhanced tolerance to abiotic stresses [10,15].

The association with AMF can improve the absorption and utilization of nutrients; therefore, it promotes balanced nutrition for plants [16]. Furthermore, these fungi play a crucial role in bestowing benefits upon the plants they interact with, including increased resistance against diseases. Research has revealed that the symbiotic relationship between arbuscular mycorrhizal fungi and plants enhances the absorption of water and nutrients, resulting in an improvement in plant development and a strengthening of resistance to environmental stressors [17].

In view of this, this study aimed to evaluate the effects of the association of *Azospirillum brasilense* (Ab-V5 strain), *Bacillus velezensis* (ZK strain), AMF, Ab-V5 + AMF and ZK + AMF with *Zea mays* seedlings under water deficiency, aiming to observe the changes induced in morphoanatomical, biochemical, and physiological traits and, consequently, the possible increase in drought tolerance.

## 2. Material and Methods

### 2.1. Biological Material and Experimental Design

The biological material used in this study included seeds of single hybrid maize DKB 290 (Monsanto), two species of PGPB belonging to *Azospirillum* and *Bacillus velezensis*, and a pool of arbuscular mycorrhizal fungus (AMF). The AMF pool was composed of spores of the *Gigaspora margarita*, *Acaulaspora morrowiae*, *Claroideoglomuse tunicatum*, and *Rhizophagus clarus* and was kindly provided by the International Glomeromycota Cultures Collection (IGCC) of the Regional University of Blumenau (FURB). The inoculation with AMF was conducted using the addition of 10 g of fresh soil inoculum containing spores, hyphas, and roots colonized in the holes made in each pot.

The bacterial species used are part of the Plant Growth-Promoting Bacteria Collection of the State University of Londrina (UEL), where they are deposited. The *Azospirillum brasilense* (diazotrophic Ab-V5 strain) is registered at the Ministry of Agriculture of Brazil for use in commercial inoculants [18]. The *Bacillus velezensis* (ZK strain) was characterized as a PGPB species and can synthesize auxins and siderophores, which are important compounds for promoting plant growth; however, this strain is not diazotrophic [19].

The inoculants were prepared according to [20]. The PGPB species were initially cultured in 5 mL of liquid DYGS medium (glucose 2 g L^−1^; peptone 1.5 g L^−1^; 2 g L^−1^ yeast extract; K_2_HPO_4_ 0.5 g L^−1^; MgSO_4_·7H_2_O 0.5 g L^−1^) in test tubes kept under orbital shaking (180 rpm) at 28 ± 2 °C for 24 h for the preparation of the preinoculum. The inoculants were prepared by the addition of the pre-inoculum (1 mL) to Erlenmeyer flasks containing 50 mL of FORM15 culture medium (glycerol 100 g L^−1^; sucrose 50 g L^−1^; yeast extract 50 g L^−1^; xanthan gum 1 g L^−1^; PVP 1 g L^−1^; Fe-EDTA 50 mM 2 mL L^−1^; MgSO_4_·7H_2_O 1 g L^−1^; NaCl 0.1 g L^−1^; KH_2_PO_4_ 4 g L^−1^; K_2_HPO_4_ 6 g L^−1^; NH_4_NO_3_ 1.5 g L^−1^) supplemented with 5 mL L^−1^ of micronutrient solution (H_3_BO_3_ 1.4 g L^−1^; ZnSO_4_·7H_2_O 1.2 g L^−1^; MnSO_4_ × H_2_O 1.18 g L^−1^; Na_2_MoO_4_·2H_2_O 1.0 g L^−1^; CuSO_4_·5H_2_O 0.04 g L^−1^), adjusted to pH 6.5 and followed by orbital shaking (180 rpm) at 28 ± 2 °C for 48 h. After the growth period, the cultures were normalized by dilution with sterile FORM15 culture medium to a final density of 1 × 10^6^ cells mL^−1^, constituting the inoculants used in the assays.

Previously to inoculation, maize seeds were surface disinfested via immersion in 95% (*v*/*v*) ethanol solution for 30 s, followed by immersion in 5% (*v*/*v*) H_2_O_2_ solution for 10 min. After, the seeds were washed six times with sterile deionized water [21]. Seeds were planted in plastic pots (400 cm^3^ volume, 10.5 cm in height, 9.5 cm in lower diameter, and 14 cm in upper diameter) filled with sterilized sand as a substrate and inoculated by applying 1 mL of the inoculant on each seed. The pots were irrigated once a week with 40 mL of the nutritive solution, which was composed of 1 mM KH_2_PO_4_, 2 mM K_2_SO_4_, 2 mM MgSO_4_, 2 mM CaCl_2_, the micronutrients described by [22], and KNO_3_ as the N source (total [N] = 4 mM).

The experiments were carried out from September to October 2015 (winter–spring). The plants were kept for 45 days in a greenhouse under natural conditions of relative humidity and temperature and 45% of total environmental photosynthetic photon flux density. Irradiance on sunny days at the greenhouse was approximately 800 µmol m^−2^ s^−1^. The average daily values ± SD of temperature, relative humidity (RH), and accumulated global solar radiation were 23.1 ± 3.1 °C, 80.2% RH ± 15.3% RH, and 15.7 ± 5.5 MJ m^−2^, respectively (data kindly provided by the Laboratory of Agrometeorology, Embrapa Soja, Londrina).

The experimental design was 6 × 1 and completely randomized with five inoculation treatments, one non-inoculated treatments (control), and all treatments in soil humidity conditions (moderate drought stress). The water deficit was applied in stage one of vegetative growth and the experiment was finalized in the vegetative stage two. The inoculation treatments consisted of a control treatment (no inoculation), two inoculation treatments with single inoculations of the strains, *A. brasilense* (Ab-V5) and *Bacillus velezensis* (ZK); two inoculations with AMF associated with Ab-V5 and ZK, and one inoculation with only AMF. The soil humidity was controlled by irrigation, with the pots kept at a moderate drought level (MD) (14% gravimetric humidity). The MD treatment was established in the last 30 days of plant growth, applying to watering plants when necessary to avoid severe drought stress during this period.

### 2.2. Growth Analysis

At the end of the experiment, the seedlings were carefully removed from the plastic pots, and the roots were washed in water. Root, shoot, and total dry weight (g) were determined after storing these organs for three days at 60 °C. After that, the root:total dry weight ratio (R:TDW) and the shoot:total dry weight ratio (S:TDW) were calculated.

### 2.3. Anatomical Analysis

Samples of the apical portion of maize adventitious roots were collected and prepared for anatomical analysis by fixation in FAA_50%_ solution (0.5 mL of formaldehyde, 0.5 mL of acetic acid, and 9 mL of 50% ethylic alcohol) for 72 h, dehydrated in ethanol series and included in methacrylate (Historesina, Leica Instruments, Heidelberg, Germany) [23]. Subsequently, the samples were sectioned in an automatic feed rotary microtome (Leica RM 2255) at the Plant Anatomy Laboratory in UFV at a thickness of 7 μm and after being stained with toluidine blue pH 4.0 [24]. The slides were mounted with Permount (SP15-500, Fisher Scientific, Fair Lawn, NJ, USA). The micromorphometric evaluations were measured using two cuts of each treatment, and in each cut, 3 measurements of tissue thickness and regions were taken. The vascular cylinder area was measured using the number of vessel elements in the metaxylem. Image Pro-Plus software (version 4.5) was used for all measurements. The observations and the digitalization of the images were performed in a photomicroscope (model Olympus CX 41, Tokyo, Japan) with a U-Photo system, in which the following parameters were determined: vascular cylinder thickness, vascular cylinder area, and the area of metaxylem vessel elements (MVEs).

### 2.4. Biochemical and Physiological Analysis

Lipid peroxidation was analyzed as a marker of oxidative stress. Freshly collected leaves (100 mg) were ground to a powder in liquid N_2_, homogenized with cold TCA (0.2%) diluted in methanol, and then centrifuged at 10,000× *g* for 5 min. The supernatant was used for the determination of the (MDA) content using the thiobarbituric acid reactive substance (TBARS) method (ex/em: 535/590 nm) [25].

To determine the activity of the enzyme nitrate reductase (NR), 150 mg of completely expanded leaves were collected and NR activity was determined in vivo based on the method proposed by [26]. Leaf samples were cut into segments and transferred to a syringe containing the reaction solution. The syringe was maintained at 28 °C under vacuum (for infiltration of the reaction solution in the tissues) in the dark and, after 40 min, an aliquot of the reaction solution was collected and the NO_2_^−^ concentration was determined with the Griess reagent [27].

The chlorophyll fluorescence was measured on the adaxial leaf surfaces using an OS1p fluorometer (Opti-Sciences, Hudson, NH, USA). The maximum quantum yield of the PSII photochemistry, expressed as *F_v_*/*F_m_* = (*F_m_* − *F*_0_)/*F_m_*, was measured at predawn (05:00–06:00 h). After 20 min of dark adaptation using FL-DC clips (Opti-Sciences, Hudson, NH, USA), the minimum fluorescence (*F*_0_) was measured using a weak modulated light, and the maximum fluorescence (*F_m_*) was determined after a 0.8-s saturating light pulse of 8250 µmol m^−2^ s^−1^.

To determine the relative water content (RWC), we collected six random samples (the last fully expanded leaf) from each treatment condition to assess the fresh weight. We determined the weight of the completely turgid sample after 24 h in the water and the dry weight according to the methods of [28]. To evaluate the RWC, we used the following equation:

$$\frac{\mathrm{Fresh}\;\mathrm{weight}-\mathrm{Dry}\;\mathrm{weight}}{\mathrm{Turgid}\;\mathrm{weight}-\mathrm{Dry}\;\mathrm{weight}}\times 100$$

### 2.5. Statistical Analysis

Seven biological replicates were used for the morphological analyses, while three were used for the biochemical, physiological, and anatomical determinations. The data were analyzed using one-way ANOVA and, when necessary, the means were compared using Tukey’s post-hoc test (*p* ≤ 0.05). Statistical analyses were performed using Statistica software version 10.0 (Statsoft Inc., Tulsa, OK, USA).

## 3. Results

### 3.1. Growth Analysis

The inoculation with ZK and ZK + AMF led to an increase in the total dry weight and shoot:total dry weight ratio of the seedlings when compared to the non-inoculated plants (control), as well as a decrease in the root:total dry weight ratio (Table 1). The ZK + AMF and Ab-V5 treatments led to an increment in the shoot or root dry weights, respectively.

### 3.2. Biochemical and Physiological Analysis

**Figure 1:**
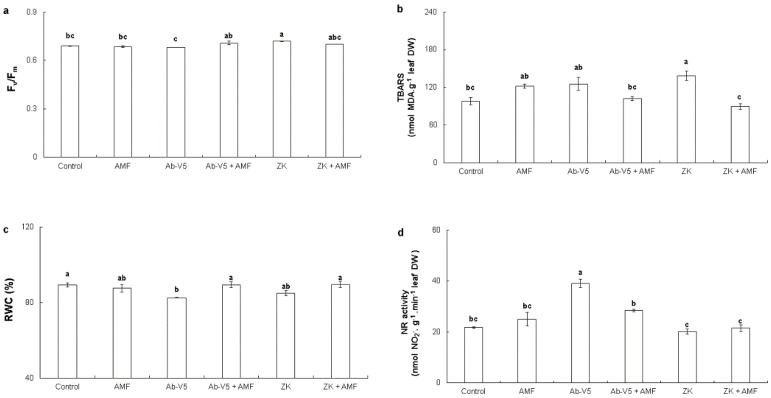
*F_v_*-*F_m_* (**a**), TBARS (**b**), RWC (**c**), and NR activity (**d**) of *Zea mays* seedlings inoculated with two species of bacteria (*Bacillus velezensis*—ZK and *Azospirillum brasilense*—Ab-V5) and arbuscular mycorrhizal fungus (AMF). Values are means ± SE (n = 3). Equal letters do not differ by Tukey’s test (*p* ≤ 0.05).

The ZK inoculation increased the *F_v_*/*F_m_* and lipid peroxidation levels of maize leaves (Figure 1a,b). Inoculation with Ab-V5 led to a reduction in RWC and an increase in NR activity in relation to the control (Figure 1c,d).

### 3.3. Anatomical Analysis

**Figure 2:**
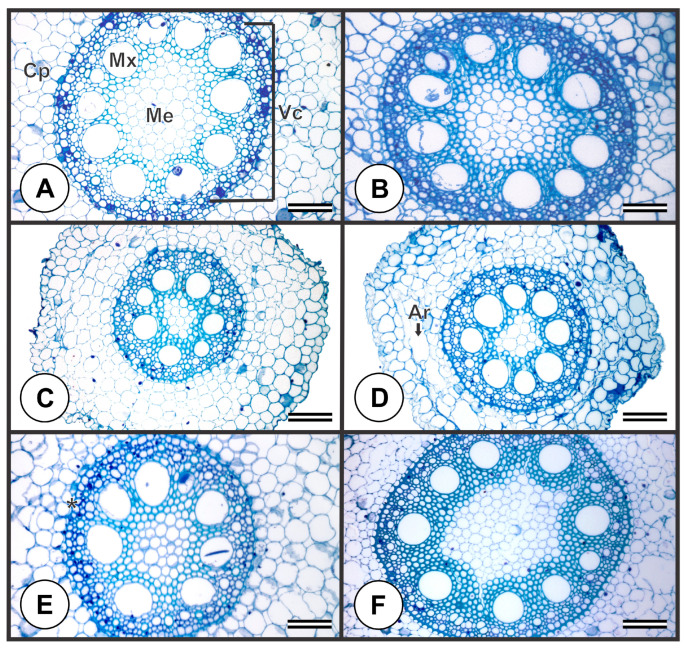
Cross-sections of roots of *Zea mays* under moderate drought and not inoculated (**A**), inoculated with AMF (**B**), Ab-V5 (**C**), Ab-V5 + AMF (**D**), ZK (**E**), and ZK + AMF (**F**). Ar: aerenchyma. Cp: cortical parenchyma. Vc: vascular cylinder. Me: medulla. Mx: metaxylem. * Endodermis with Striae of Caspary. Bars = 100 μm.

Compared to non-inoculated plants (Figure 2A), AMF and Ab-V5 inoculation (Figure 2B,C) led to an increase in the area of the MVE (Table 2). AMF, ZK, and ZK + AMF inoculation resulted in a thicker vascular cylinder (Figure 2B,E,F) (Table 2). For the plants inoculated with ZK and ZK + AMF (Figure 2E,F), an increase in the vascular cylinder area was observed.

## 4. Discussion

The association with PGPB and AMF led plants to develop biochemical and morphological mechanisms that may improve their tolerance to drought. It is well known that these microorganisms can provide essential nutrients or facilitate their access to the plant, as well as confer greater tolerance for plants against biotic and abiotic stresses [29,30]. Inoculation with *Azospirillum* or *Bacillus* can increase the plant biomass, nutrient uptake, and root exudations as an effect of alterations to the plant’s regular hormonal balance, which follows the colonization of plant tissues by the inoculant bacteria and retrofeeds this event [15]. In this sense, the increment in the total dry weight, shoot dry weight, and shoot:total dry weight evidences the greater compatibility of the ZK *Zea mays* under MD. Besides that, in *Zea mays* seedlings subjected to different nitrogen availabilities, the association with *Bacillus* sp. (later characterized as *B. velezensis*–strain ZK) positively influenced the net photosynthetic rate, maximum quantum efficiency of photosystem II, length, and number of vessels in the root metaxylem [15]. Similar results were already observed in pepper plants inoculated with *Bacillus* sp. [31].

Therefore, despite the increased lipid peroxidation in the present study, and as observed by [31] in *Capsicum chinense*, our results showed that the ZK strain association improved chlorophyll fluorescence, suggesting more reaction centers and higher light efficiency use. Thus, the quinone acceptor (Qa) is highly oxidized; moreover, its excitation energy is utilized in electron transport, leading to high H^+^ release in the thylakoid lumen, which causes an increment in ATP and NADPH synthesis, that are employed in C assimilation in the Calvin cycle, improving maize growth as observed in the higher TDW compared to no inoculated.

Ref. [32] verified that water deficiency disrupted the electron transport in photosynthetic apparatus; however, AMF symbiosis was able to mitigate the adverse influence of drought stress on the PSII reaction center in maize plants. In the present study, the association with AMF did not mitigate the negative impact of drought on photosynthetic efficiency, as verified by the *F_v_*/*F_m_* values. However, the inoculation with ZK increased the *F_v_*/*F_m_* compared to the control, indicating a lower need for thermal dissipation under MD by plants inoculated with this bacterial strain [29,33].

Moreover, the root dry weight indicates that the Ab-V5 strain interferes with root growth. This effect may be attributed to the effects of phytohormones induced in the vegetal tissues by the PGPB. Moreover, this can be due to the synthesis of components responsible for stimulating root growth, including indole-3-acetic acid (IAA), gibberellins, and cytokinins by the PGPB [34]. Besides that, ZK strain inoculation results in increased TDW and shoot:total dry weight ratio, which may be related to the producer of indolics [19].

In the present study, the inoculation with Ab-V5 also positively influences NR activity, as already observed in leaves of lettuce, rice, other cereals, and native trees inoculated with PGPB [10,35]. This response may be due to the increment in the absorption of nitrate (NO_3_^−^) since the nitrate is the activating substrate for NR and has important action in the synthesis of the RNAm of the NR [10,36]. Ref. [37] verified that an *Azospirillum brasilense* strain induced the shoot NR activity in wheat plants under a water deficit, accompanied by increased amino acid content. They concluded that *Azospirillum* inoculation might play an important role in protein biosynthesis at low soil moisture. Thus, despite the reduction in the RWC in the Ab-V5 inoculation, better assimilation of the NO_3_^−^ could have induced the increase in root dry weight in this study. Therefore, this increased capacity for N assimilation can enable the host plant to be more tolerant to drought conditions [9].

Anatomical changes induced by the inoculation with PGPB and AMF in this study (Table 2) emphasize the role of these microorganisms in promoting alterations that are relevant to plant growth [15]. In this sense, the increase in these parameters can lead to better shoot hydraulic conductivity once the modification of vessel size has an important role in the enhanced tolerance to drought since that can increase water and nutrients transport from the roots to the leaves [38,39,40].

The xylem vessels of maize plants can suffer embolism and cavitation under drought [41,42]. Therefore, anatomical changes of root tissues are relevant during water deficit, especially because they influence water and ion flow under these conditions [43]. In a previous study, the inoculation with *Bacillus* sp. (ZK strain) increased the vascular cylinder thickness and the area of metaxylem vessel elements in maize plants [15]. Here, under MD, the metaxylem area did not differ between the control and plants inoculated with *Bacillus* sp.; however, the vascular cylinder area showed a strong increase of 124% and 173% in plants inoculated with ZK and ZK + AMF, respectively.

The synthesis of the auxin via PGPB used in this study could induce the higher differentiation of the procambium cells to vessels [15,19]. It is already known that the ZK strain used in this study can produce indolic compounds [19], and the auxin, which is an indolic compound, induces differentiation in plant tissues; thus, the synthesis of auxin by PGPB probably causes the effect observed in xylem vessels [40]. Moreover, the microorganism association can also enhance cell wall elasticity and apoplastic water content of the coleoptile, which may lead to an increase in drought tolerance [44]. The increment in the area and number of MVE can also have an important role in drought tolerance since, as observed by [45], these plants can be more efficient in conducting water. Thus, the vascular development changes in maize may be related to the synthesis of auxin by the PGPB used in this study.

This study lays the groundwork for understanding maize response to moderate drought in association with PGPB, with implications for more severe drought scenarios. Identified tolerance morpho-physiological and biochemistry mechanisms, such as increased total dry weight and nitrate reductase activity, suggest potential applicability under extreme drought conditions. The observed resilience with the PGPB association indicates promising prospects for mitigating agricultural losses due to climate change.

In recent years, RNA sequencing studies in host plants such as rice and sugarcane during associations with PGPB (such as *Azospirillum*, *Herbaspirillum*, and *Burkholderia*) have identified plant genes potentially involved in aspects of the association, such as recognition and nutrient transport. Most of these studies have been conducted in roots and have revealed genes related to flavonoid biosynthesis, hormones, and transporters, which are promising targets for future research [46,47,48]. However, the study suggests limitations in using AMF under severe drought, highlighting the need for further research to optimize their efficacy through strain selection and combination with other microorganisms.

## 5. Conclusions

The association with ZK changed the morphoanatomical and physiological parameters, and the Ab-V5 inoculation affected NR activity and morphoanatomical parameters. Therefore, these microorganisms can be an alternative to contribute to the reduction in the losses in maize crops under drought. Still, the AMF inoculation did not lead to great changes in maize that can be related to drought tolerance.

## Figures and Tables

**Table 1:** Morphological parameters of *Zea mays* seedlings inoculated with two species of bacteria (*Bacillus velezensis*—ZK and *Azospirillum brasilense*—Ab-V5) and arbuscular mycorrhizal fungus (AMF). Values are means ± SE (n = 7). Equal letters in the columns indicate values that do not differ by Tukey’s test (*p* ≤ 0.05). R: root. S: shoot. TDW: total dry weight.

Treatments	R Dry Weight (g)	S Dry Weight (g)	TDW (g)	R:TDW	S:TDW
Control	0.33 ± 0.02 b	0.42 ± 0.11 b	0.75 ± 0.11 c	0.44 ± 0.04 a	0.56 ± 0.04 b
AMF	0.35 ± 0.01 ab	0.56 ± 0.07 ab	0.91 ± 0.08 bc	0.39 ± 0.02 ab	0.61 ± 0.02 ab
Ab-V5	0.42 ± 0.01 a	0.55 ± 0.10 ab	0.97 ± 0.10 abc	0.46 ± 0.05 a	0.54 ± 0.05 b
Ab-V5 + AMF	0.41 ± 0.02 ab	0.45 ± 0.11 b	0.84 ± 0.14 bc	0.38 ± 0.03 ab	0.62 ± 0.03 ab
ZK	0.38 ± 0.01 ab	0.75 ± 0.05 ab	1.14 ± 0.04 ab	0.33 ± 0.02 b	0.67 ± 0.02 a
ZK + AMF	0.36 ± 0.03 ab	0.87 ± 0.06 a	1.27 ± 0.06 a	0.33 ± 0.01 b	0.67 ± 0.01 a

**Table 2:** Anatomical parameters of *Zea mays* seedlings inoculated with two species of bacteria (*Bacillus velezensis*—ZK and *Azospirillum brasilense*—Ab-V5) and arbuscular mycorrhizal fungus (AMF). Values are means ± SE (n = 3). Equal letters in the columns indicate values that do not differ by Tukey’s test (*p* ≤ 0.05). VCT: vascular cylinder thickness. VCA: vascular cylinder area. MVE: metaxylem vessel elements.

Treatments	VCT (μm)	VCA (mm^2^)	Area MVE (μm^2^)
Control	272 ± 13.3 c	0.236 ± 0.025 c	7667.33 ± 327.79 c
AMF	415 ± 15.89 a	0.436 ± 0.058 abc	13,249.38 ± 584.54 ab
Ab-V5	254 ± 24.9 c	0.309 ± 0.110 bc	15,604.77 ± 1664.67 a
Ab-V5 + AMF	299 ± 8.92 bc	0.289 ± 0.017 bc	9252.42 ± 1054.67 bc
ZK	372 ± 15.0 ab	0.522 ± 0.046 ab	11,630.35 ± 973.36 abc
ZK + AMF	444 ± 33.0 a	0.636 ± 0.061 a	10,173.82 ± 300.85 bc

## Data Availability

Data is contained within the article.
